# ROSC rates and live discharge rates after cardiopulmonary resuscitation by different CPR teams - a retrospective cohort study

**DOI:** 10.1186/s12871-017-0457-5

**Published:** 2017-12-04

**Authors:** Tak Kyu Oh, Young Mi Park, Sang-Hwan Do, Jung-Won Hwang, In-Ae Song

**Affiliations:** 1 Interdepartment of Critical Care Medicine, Seoul National University Bundang Hospital, 82, Gumi-ro 173 Beon-gil, Bundang-gu, Seongnam-si, Gyeonggi-do 463-707 South Korea; 2 Department of Anesthesiology and Pain Medicine, Seoul National University Bundang Hospital, 82, Gumi-ro 173 Beon-gil, Bundang-gu, Seongnam-si, Gyeonggi-do 463-707 South Korea; 3 Medical Research Collaborating Center, Seoul National University Bundang Hospital, 82, Gumi-ro 173 Beon-gil, Bundang-gu, Seongnam-si, Gyeonggi-do 463-707 South Korea

**Keywords:** Hospital, Resuscitation, Intensive care

## Abstract

**Background:**

Previous studies have reported that the quality of cardiopulmonary resuscitation (CPR) is closely associated with patient outcomes. The aim of this study was to compare patient CPR outcomes across resident, emergency medicine, and rapid response teams.

**Methods:**

The records of patients who underwent CPR at the Seoul National University Bundang Hospital from January 1, 2013 to December 31, 2016 were analyzed retrospectively. Return of spontaneous circulation, 10- and 30-day survival, and live discharge after return of spontaneous circulation were compared across patients treated by the three CPR teams.

**Results:**

Of the 1145 CPR cases, 444 (39%) were conducted by the resident team, 431 (38%) by the rapid response team, and 270 (23%) by the emergency medicine team. The adjusted odds ratios for the return of spontaneous circulation and subsequent 10-day survival among patients who received CPR from the resident team compared to the rapid response team were 0.59 (*P* = 0.001) and 0.71 (*P* = 0.037), respectively. There were no significant differences in the 30-day survival and rate of live discharge between patients who received CPR from the rapid response and resident teams; likewise, no significant differences were observed between patients who received CPR from the emergency medicine and rapid response teams.

**Conclusions:**

Patients receiving CPR from the rapid response team may have higher 10-day survival and return of spontaneous circulation rates than those who receive CPR from the resident team. However, our results are limited by the differences in approach, time of CPR, and room settings between teams.

## Background

For patients with cardiac arrest, outcomes and survival depend greatly on timely and effective cardiopulmonary resuscitation (CPR) by experienced healthcare workers [1]. Improving patient outcomes by improving the quality of CPR has been crucial in the fields of critical care medicine and resuscitation [2, 3].

In an effort to improve CPR outcomes, many hospitals have designated professional CPR teams [4]; however, the most effective resuscitation models for improving outcomes remain controversial [5, 6]. Several studies have reported that having either a medical emergency team or a rapid response (RR) team designated for CPR could increase the quality of CPR [7, 8]. Unfortunately, most of these studies have been limited in that they compared patients “before” and “after” implementation of either RR or medical emergency team [7, 8].

Seoul National University Bundang Hospital (SNUBH) is a tertiary academic hospital that implemented a part-time RR team in October 2012 to conduct in-hospital CPR covering 47.6% of the week [9], while the on-call residents were responsible for the remaining 52.4% of the week. Separate from these teams, the emergency medicine (EM) team responded to all of the out-of-hospital CPR cases, as well as the in-hospital CPR cases occurring in the emergency department. The aim of this study was to compare patient CPR outcomes across the RR, resident, and EM teams. Specifically, we wanted to examine whether CPR performed by the RR team yielded superior patient outcomes than that performed by the EM and resident teams.

## Methods

### Study design

This was a retrospective cohort study approved by the Institutional Research Board at SNUBH (B-1704/390–102, Approval Date: March 29, 2017). Because this was a retrospective review of patient medical records, the requirement for informed consent was waived. The study examined records of patients (both adult and pediatric) who had been administered CPR between January 1, 2013 and December 31, 2016. Infants younger than 2 years, and patients with incomplete records or missing data, were excluded from the study. The STROBE guidelines for reporting on observational cohort studies were followed.

### Clinical setting

The SNUBH is a tertiary academic hospital. As of May 2017, it had a total of 1164 ward- and 102 intensive care unit (ICU)-beds. Since 2003, an electronic medical record system has been used to manage all medical records, including CPR data.

### CPR system in SNUBH

Since January 2013, the CPR system of SNUBH has been divided into three distinct teams according to the time and place of CPR administration. The first is the RR team that operates Monday–Friday from 7 AM–10 PM, and on Saturdays from 7 AM–12 PM, in order to respond to all in-hospital CPR cases except for those occurring in the emergency department and operating rooms [9]. This team includes 12 multi-disciplinary intensivists (belonging to internal medicine, anesthesiology, emergency medicine, and thoracic surgery) and four highly qualified nurses with special training for CPR and a work experience of more than 5 years in the ICU.

The second team is the resident team that operates Monday–Friday from 10 PM–7 AM, and from 12 PM on Saturday to 7 AM on Monday (when the part-time RR team is not on duty) in order to respond to all in-hospital CPR cases, except those occurring in the emergency department and operating rooms. The team consists of on-call residents (from the emergency and medical ICUs), typically in their 2nd and 3rd years of residency, and nurses (from the medical wards and ICU).

The third team is the EM team that responds to all CPR cases occurring in the emergency department regardless of day of the week, and also all of the out-of-hospital CPR cases. The EM team includes EM staff physician, EM residents, EM technicians, and EM nurses.

### Activation of CPR teams in SNUBH

In SNUBH, anytime a patient is in need of CPR, it is announced throughout the hospital using a broadcasting system (as “Code Blue”). This system for announcement is used for all in-hospital CPR cases that occur in a ward or ICU. The respective team, then, according to its designated operating schedules (daytime for the RR team, nights and weekends for the resident team), goes to the patient to perform CPR. CPR in the emergency room, on the other hand, is carried out by the EM team without announcement through the broadcasting system. In addition, the EM team is promptly informed of the out-of-hospital cardiac-arrest cases by the Emergency Medical Service (EMS), upon which they systematically respond to administer CPR to the patient.

### Measurements and outcomes

We collected data on gender, age, height (cm), bodyweight (kg), duration of CPR (min), time from cardiac arrest to CPR (min), attempts for endotracheal intubation, artificial airway use, return of spontaneous circulation (ROSC) rate, the Charlson comorbidity score, and survival status of the in-hospital CPR cases. The CPR duration was defined as the length of time between the initial mask ventilation or chest compression and ROSC or pronouncement of death. In the case of in-hospital CPR, time from cardiac arrest to CPR initiation was defined as the length of time between detection of arrest to the initial mask ventilation or chest compression. For out-of-hospital CPR cases, time from cardiac arrest to CPR initiation was defined as the length of time between hospital arrival and initial mask ventilation or chest compression. If CPR was performed during arrival at the emergency department, or if CPR commenced within a minute of detection (in cases of in-hospital CPR), time from cardiac arrest to CPR initiation was defined as 0 min. All medical records were reviewed by a medical record technician from the SNUBH informatics team, who was blinded to the study’s purpose and had no potential conflict of interest.

The primary outcome of the study was the rate of ROSC among patients who received CPR, stratified by the team that provided CPR. The secondary outcomes were the rates of 10- and 30-day survival, and live discharge after ROSC, stratified by the team that provided CPR. Additionally, the rates of ROSC, 10- and 30-day survival, and live discharge post-ROSC were compared between the CPR cases in the surgical and non-surgical departments.

### Statistical method

We analyzed the patients by the survival status and by the three teams that had administered the CPR. First, using the entire cohort, we analyzed the distribution of the 10-day survival and ROSC rates according to each team. Then, among the ROSC survivors, we also analyzed the distribution of 10-day survivors, 30-day survivors, and patients who were alive at the time of discharge. Next, the chi-square test was conducted to determine whether any of the three teams were risk factors for the rate of ROSC, 10-day survival, 30-day survival, and live discharge.

We conducted the Cochran-Mantle-Hensel test after application of the chi-square test, in order to see if the meaningful outcome of the chi-square test was controlled and matched with the entire cohort (2013–2016); the aim was to control for yearly variation to see if there remained an association between each team and the survival rate. In addition, we conducted a post-hoc power analysis to determine the validity of the chi-square test used to test the hypothesis (difference in ROSC rates) in our study; the sample size (*n* = 1145) of our study was sufficient for a power of 91%.

Finally, based on the patient characteristic analysis, we developed a multivariate logistic regression model of the four outcomes (ROSC, 10-day survival, 30-day survival, and live discharge) that were adjusted for the patients’ age, weight, and gender. *P*-values ≤0.05 were used to determine statistical significance. The statistical analyses were performed, and graphics were generated, using the open-source statistical software R, version 3.3.2 (http://www.r-project.org) with ggplot2 packages and Stata software, version 14 (StataCorp LP, College Station, TX).

## Results

During the study period, there were a total of 1148 CPR cases. Three cases were excluded due to incomplete data; of the remaining 1145 CPR cases, 444 (39%) were conducted by the resident team, 431 (38%) by the RR team, and 270 (23%) by the EM team. The baseline characteristics of the patients attended by the three teams are shown in Table 1. The mean Charlson comorbidity scores did not significantly differ among patients treated by the three teams (RR team: 2.61 ± 1.32; resident team: 2.58 ± 1.06; EM team: 2.69 ± 1.35; *P* = 0.512). The outcomes of CPR according to the variables are shown in Table 2.

**Table 1:** Characteristics of patients treated by the three CPR teams (RR, Resident, and EM teams)

			Teams			*P*-value
			RR *n* = 431	Resident *n* = 444	EM *n* = 270	
Age (years)		Mean	65.69	67.15	66.88	0.368
		SD	15.54	16.39	15.97	
Gender	Male	N	258	271	157	0.747
		%	37.6	39.5	22.9	
	Female	N	173	173	113	
		%	37.7	37.7	24.6	
Height (cm)		Mean	158.21	157.70	159.47	0.698
		SD	23.89	24.66	22.79	
Weight (kg)		M	66.53	65.49	64.51	0.198
		SD	13.42	14.08	13.29	
Defibrillation during CPR		N	104	93	39	0.008
		%	44.1	39.4	16.5	
Type of arrest	Cardiac arrest	N	348	360	224	0.745
		%	37.3	38.6	24.0	
	Respiratory arrest	N	83	84	46	
		%	39.0	39.4	21.6	
CPR start after cardiopulmonary arrest (min)		Mean	0.60	0.50	0.45	0.357
		SD	1.647	1.359	1.262	
Placement of advanced airway		N	194	188	122	0.661
		%	38.5	37.3	24.2	
Attempt for endotracheal intubation	No intubation	N	237	255	148	0.174
		%	37.0	39.8	23.1	
	First attempt	N	180	161	109	
		%	40.0	35.8	24.2	
	>Two attempts	N	14	28	13	
		%	25.5	50.9	23.6	
CPR time (min)		M	15.35	16.45	14.90	0.522
		SD	19.841	20.558	15.347	
Charlson comorbidity index		M	2.61	2.58	2.69	0.512
		SD	1.32	1.06	1.35	

RR: Rapid Response; EM: Emergency Medicine; SD: Standard Deviation; CPR: Cardiopulmonary Resuscitation

**Table 2:** Outcomes of CPR according to variables

Variable	ROSC (*n* = 1145)			10-day survival (*n* = 1145)			30-day survival (*n* = 1145)			Live discharge (*n* = 1145)		
	Yes	No	*P*	Yes	No	*P*	Yes	No	*P*	Yes	No	*P*
Gender (%)												
Male Female	62.8 37.2	53.6 46.4	0.003	64.1 35.9	57.8 42.2	0.039	63.8 36.2	58.4 41.6	0.093	61.4 38.6	59.5 40.5	0.579
Age (years)	65.5 (15.8)	68.8 (16.1)	0.001	64.21 (0.84)	67.7 (15.7)	<0.001	63.6 (16.8)	67.7 (15.5)	<0.001	63.2 (17.2)	67.50 (15.5)	<0.001
Duration of CPR (min)	16.0 (20.3)	15.0 (16.5)	0.417	14.0 (16.2)	16.5 (20.5)	0.037	13.4 (15.2)	16.6 (20.4)	0.012	13.4 (15.2)	16.3 (20.1)	0.029
Height (cm)	158.9 (24.1)	156.6 (23.8)	0.178	159.2 (24.8)	157.7 (23.5)	0.178	158.5 (26.3)	158.1 (23.0)	0.848	158.8 (24.8)	158.0 (23.8)	0.649
Weight (Kg)	66.6 (13.7)	63.5 (13.3)	<0.001	67.8 (14.2)	64.6 (13.3)	<0.001	67.7 (14.7)	64.9 (13.2)	0.002	66.8 (13.2)	65.4 (13.8)	0.138
CPR start from cardiac arrest (min)	0.58 (1.56)	0.43 (1.19)	0.124	0.55 (1.52)	0.52 (1.42)	0.758	0.55 (1.54)	0.52 (1.42)	0.816	0.52 (1.50)	0.53 (1.44)	0.888
Attempts for endotracheal intubation (%)	1: 37.9 2: 4.3 3: 0.5 4: 0.4	1: 42.5 2: 3.63 3: 0.3 4: 0.0	0.442	1: 34.6 2: 3.9 3: 0.3 4: 0.5	1: 41.7 2: 4.2 3: 0.5 4: 0.1	0.106	1: 33.7 2: 3.8 3:0.3 4:0.3	1: 41.5 2: 4.2 3: 0.5 4: 0.2	0.148	1: 35.4 2: 3.15 3: 0.39 4: 0.0	1: 40.4 2: 4.4 3: 0.5 4: 0.3	0.358
No. Advanced Airway (%)	56.9	53.6		60.7	53.5		62.0	53.6		61.0	54.4	

The data were represented as mean (standard deviation)

CPR: Cardiopulmonary Resuscitation; ROSC: Return of Spontaneous Circulation

### ROSC rates, 10- and 30-day survival, and live discharge after ROSC

**Fig. 1:**
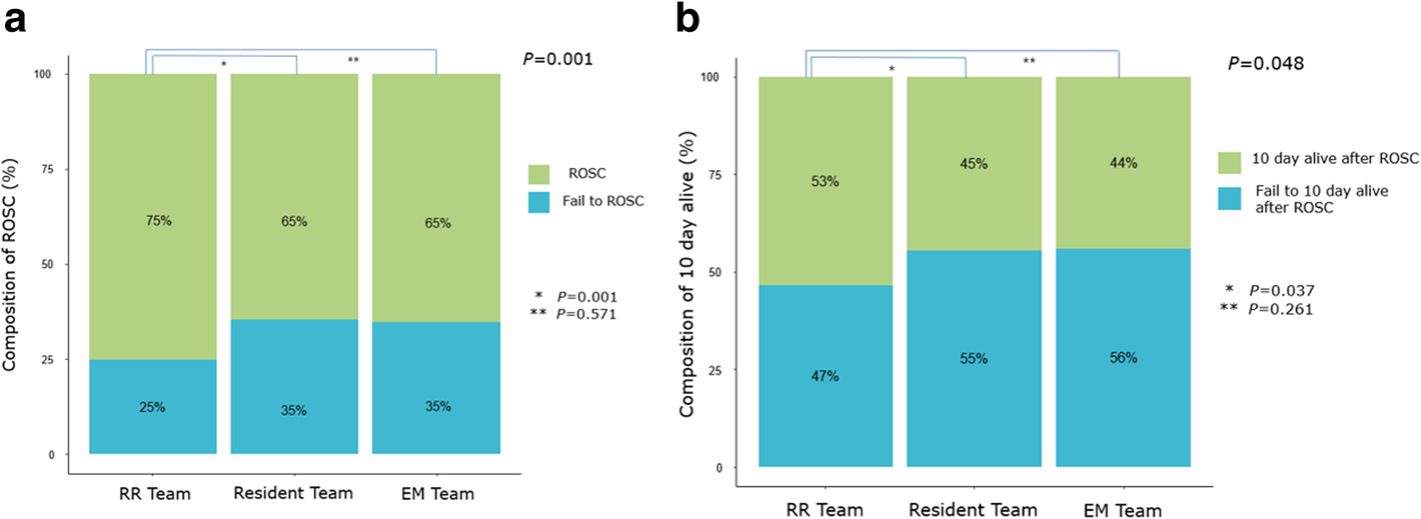
Rate of ROSC (**a**) and 10-day survival (**b**) between the three teams (RR team, resident team, EM team). ROSC, return of spontaneous circulation; RR, rapid response; EM, emergency medicine. The Chi-square revealed that the rate of ROSC (*P* = 0.001) and the 10-day survival rate (*P* = 0.048) were higher in the RR team than in the resident and EM teams. The multivariate logistic regression model was adjusted for the patients’ age, weight, and gender by adding to the team. (*RR team versus resident team, **RR team versus EM team)

**Fig. 2:**
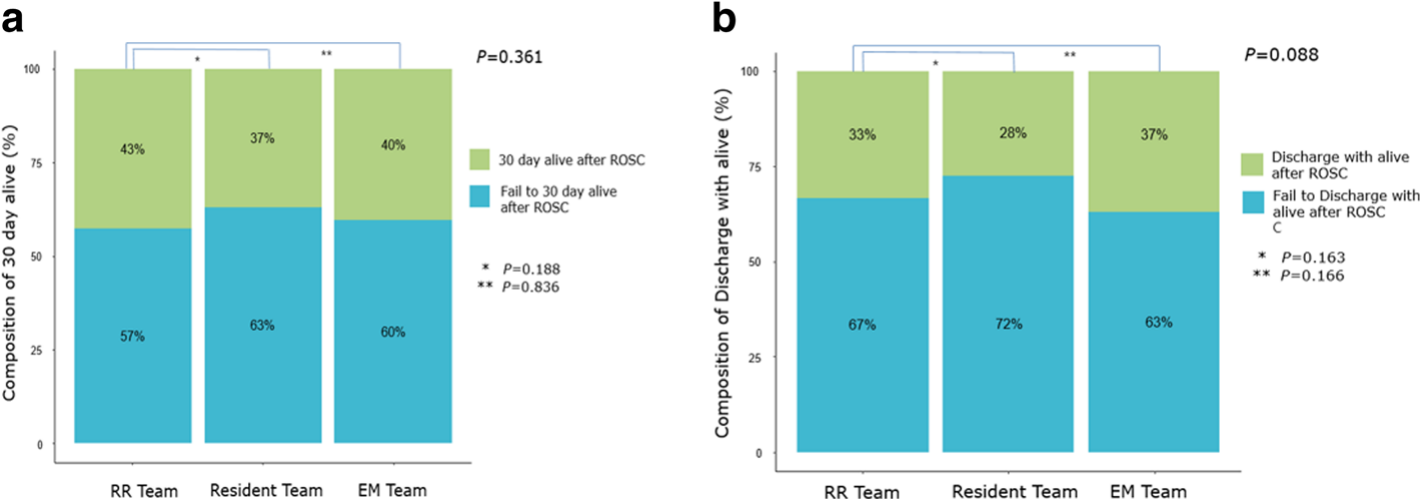
Composition of 30-day survival (**a**) and live discharge (**b**) among the three teams (RR team, resident team, EM team). RR, rapid response; EM, emergency medicine. The Chi-square revealed that the rate of 30-day survival (*P* = 0.361) and live discharge (*P* = 0.088) were did not vary significantly among the CPR teams. The multivariate logistic regression model was adjusted for the patients’ age, weight, and gender by adding to the team. (*RR team vs. resident team, **RR team vs. EM team)

The ROSC rate among patients treated by the RR team was 75% (324/431); this was significantly higher than that observed among the patients treated by the resident team (65%; 287/444) or the EM team (65%; 176/270) *P* = 0.001; (Fig. 1a). The 10-day survival rate among the patients who achieved ROSC was 53% (173/324) in those treated by the RR team; this was significantly higher than that observed among patients treated by the resident (45%, 128/287) or the EM (44%, 78/176) teams (Fig. 1b, *P* = 0.048). The 30-day survival among those who achieved ROSC did not vary significantly among the patients treated by the three CPR teams (RR team: 43%, 138/324; resident team: 37%, 106/287; EM team: 40%, 71/176; *P* = 0.36; Fig. 2a). Similarly, the rate of live discharge after ROSC did not vary significantly among patients treated by the three CPR teams (RR team: 33%, 108/324; resident team: 28%, 79/287; EM team: 37%, 65/176; *P* = 0.087; Fig. 2b).

### Adjusted odds ratio comparing the RR, resident, and EM teams

Based on Table 2, we performed multiple regression analysis with variables including gender, age, and weight, which have been found to have a significant effect on the outcome of CPR. Seventy-three patients were excluded from multiple regression analysis due to missing data (accurate bodyweight); the remaining 1072 patients were included in the analysis (Table 3). A multivariate logistic regression was performed controlling for age, height, weight, and gender of the patients (Table 3). There were no significant differences in the rates of ROSC, 10- and 30-day survival, and live discharge between patients treated by the RR and EM teams. In contrast, the adjusted odds ratios for ROSC and 10-day survival when patients were treated by the resident team were 0.59 (*P* = 0.001) and 0.71 (*P* = 0.037), respectively (Table 3). However, there were no significant differences between the patients of the RR and resident teams with respect to the 30-day survival and live discharge rates (*P* > 0.05).

**Table 3:** Multivariate logistic analysis for outcome of CPR

Variable	ROSC (*n* = 1072)			10-day survival (*n* = 769)			30-day survival (n = 769)			Live discharge (n = 769)		
	Adjusted OR	95% CI	*P* value	Adjusted OR	95% CI	*P* value	Adjusted OR	95% CI	*P* value	Adjusted OR	95% CI	*P* value
CPR Team												
RR	ref.			ref.			ref.			ref.		
Resident	0.59	0.42–0.78	0.001	0.71	0.48–0.94	0.037	0.80	0.53–1.06	0.188	0.78	0.51–1.05	0.163
EM	1.12	0.68–1.57	0.571	0.80	0.50–1.11	0.261	1.04	0.64–1.44	0.836	1.32	0.80–1.85	0.166
Age (years)	0.99	0.98–0.99	0.007	0.99	0.98–1.00	0.027	0.99	0.98–0.99	0.005	0.98	0.98–0.99	0.002
Weight (kg)	1.01	1.00–1.02	0.032	1.01	1.00–1.02	0.121	1.01	0.99–1.01	0.278	1.00	0.99–1.01	0.966
Gender												
Male	ref.			ref.			ref.			ref.		
Female	0.91	0.63–1.18	0.538	1.006	0.67–1.34	0.972	1.022	0.68–1.37	0.896	1.13	0.73–1.53	0.492

CPR: Cardiopulmonary Resuscitation; ROSC: Return of Spontaneous Circulation; OR: odds ratio; CI: confidence interval; Ref: Reference

### CPR outcomes in surgical versus non-surgical department

**Fig. 3:**
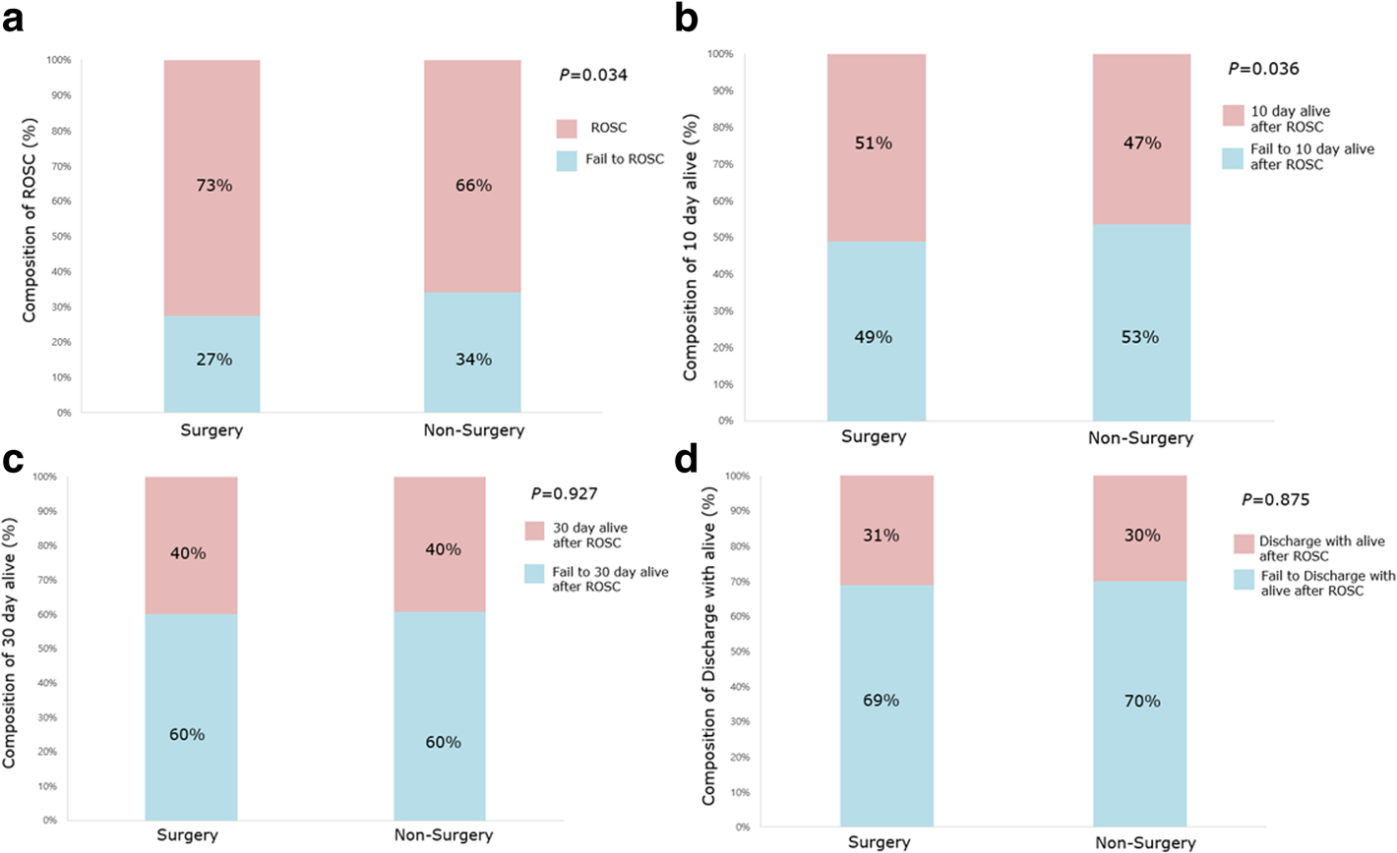
Composition of ROSC (%), (**a**) 10-day survival (%) (**b**), 30-day survival (%), (**c**), and live discharge (%), (**d**) between patients in non-surgery departments and surgery departments. ROSC, return of spontaneous circulation. The Chi-square test was conducted to determine whether the two departments (surgical vs. non-surgical) served as risk factors for ROSC, 10-day survival, 30-day survival, and live discharge

We found a significantly higher ROSC rate in the surgical department than in the non-surgical department for ward patients who had received in-hospital CPR (excluding patients who received CPR from the EM team) (*P* = 0.034, Fig. 3a). Similarly, the 10-day survival among the ROSC patients was higher in the surgical group than in the non-surgical group (51%, 188/368 vs. 47%, 113/243; *P* = 0.0366; Fig. 3b). There were no significant differences in the 30-day survival and live discharge rates between the two departments (30-day survival: 40% [147/367] vs. 40% [96/244], *P* = 0.927, Fig. 3c; live discharge rate: 31% [170/424] vs. 30% [73/287], *P* = 0.875, Fig. 3d).

## Discussion

In the present study, we found that only the ROSC rates and 10-day survival after ROSC varied between patients who had received CPR from the RR and resident teams from 2013 through 2016. Our findings suggest that the RR team was able to improve patient CPR outcomes by delivering better quality CPR than the resident team that was composed of trainees. Further, while the CPR outcomes between those treated by the EM and RR teams were not significantly different, patients that received CPR in the surgical department had higher rates of ROSC and 10-day survival than those that received it in the non-surgical department.

The first important point to consider while interpreting our findings is whether the difference observed in ROSC rates between patients that were administered CPR by the RR and resident teams was due to the difference in their operating times, and not due to the difference in the proficiency of the teams. Considering the similar nurse-to-patient ratios during the daytime and nighttime in SNUBH, it is unlikely that the occurrence of sudden cardiopulmonary arrests in patients was detected late during the nighttime. However, it is likely that the optimal performance of the resident team was affected because there may have been more fatigue or carelessness in nighttime or during weekends.

The differences in CPR outcomes between the RR and the resident teams might be attributed to either patient-related factors that we were unable to control for, or effects occurring during the night and over weekends. Previous studies have reported that hospitals tend to attend to patients with a greater severity of illness at night; hence, the in-hospital CPR conducted during the night yielded lower ROSC rates [10]. Further, because these studies [11, 12] analyzed the nighttime or weekend effects within the same CPR system, it is likely that patient-related factors and fatigue levels of the medical staff contributed to the lower ROSC rates.. In our study, it is unlikely that severity of illness contributed to the differences in the CPR outcomes; this is supported by the non-significant differences in the Charlson comorbidity scores between the three teams. However, since the effects of the time of the day and day of the week (nighttime and weekend effects) were not considered in this study, a follow-up study investigating these factors would be beneficial.

Second, when interpreting our findings, it is important to note that the EM team has been primarily responsible for CPR for out-of-hospital cardiac arrests. In general, out-of-hospital cardiopulmonary arrests are known to be worse than in-hospital cardiopulmonary arrests [13, 14]. Furthermore, the initial CPR was likely to be provided by the Public EMS team rather than the EM team from the hospital. As a result, the incidence of defibrillation and the placement of an advanced airway were fewer for patients treated by the EM team compared to those for in-hospital cardiac arrest patients treated by the RR or resident teams. Nevertheless, the initial outcome of CPR (rate of ROSC) administered by the EM team for the out-of-hospital cardiopulmonary arrests did not differ from that of CPR administered by the RR team for in-hospital cardiopulmonary arrests in covariates-adjusted multiple regression analysis (Table 3). This result shows the superiority compared to other teams of the systematic CPR implemented by the combination of the public EMS system and the EM team. However, considering that the EM team is not generally responsible for CPR for in-hospital cardiopulmonary arrest, further study is needed to investigate the quality of CPR provided by the EM team.

Other potential confounding factors in the present study were the numbers of individuals who performed CPR, and the numbers of CPR leaders. It is well known that CPR performed immediately following detection of cardiac arrest contributes to better patient outcomes [15]. However, we did not find a significant association between CPR outcomes and the length of time between detection of cardiac arrest and the CPR initiation. This is likely attributed to the fact that most of the CPR cases examined in this study had CPR performed within a minute of detection of cardiac arrest. As for the number of staff members performing CPR, an accurate comparison between the three teams would be difficult due to the retrospective nature of this study; however, all three teams in the study were designed to have at least five individuals participating in CPR implementation. Therefore, it is not likely that the number of staff members per team exerted any decisive influence on the CPR outcomes.

Furthermore, the medical personnel in charge of leading the CPR could potentially play a more decisive factor on the outcomes of the three teams. An experienced SNUBH intensivist (anesthesiologist, pulmonologist, thoracic surgeon, or emergency physician) is typically put in charge of leading the CPR for the RR team [9]. However, for the resident team, an on-call resident, in the second or third year of residency, is typically put in charge of leading the CPR; this introduces an experience gap between the RR and resident teams. Similarly, an experienced physician typically leads the EM team’s CPR, likely contributing to the high-quality CPR observed from that team. A previous study reported that having an experienced medical doctor as a CPR team leader can improve ROSC rates [16]. The presence of highly-trained nurses in the RR team, who generally have more experience than regular nurses, and the presence of emergency medical technicians in the EM team, likely contributed to higher-quality CPR, due to more organized role allocation and effective performance in these teams [17].

Finally, better patient outcomes were observed in the surgery department, regardless of which team (RR or resident team) administered in-hospital CPR. Cardiac arrest among patients in surgery departments are likely to be related to postoperative complications [18]. However, because the present study did not compare the characteristics of in-hospital CPR patients between surgical and non-surgical departments, it is highly likely that these characteristics had an impact on the CPR outcomes. Thus, more research is needed to determine the factors influencing the differences in patient outcomes between those receiving CPR in surgical and non-surgical departments.

This study was subject to several limitations. As this was a retrospective study, bias was inevitable. The time of day during which CPR was performed differed between the RR and resident teams; and nighttime and weekend effects could not be considered. Additionally, since the EM team’s CPR was limited to the emergency department, the emergency room settings may have influenced outcomes; further, records detailing how many individuals performed the CPR or the timing of defibrillation were unavailable. Finally, assessment of the EM team’s CPR quality via a direct comparison of parameters with the RR and resident teams was limited because the EM team responded mostly to out-of-hospital CPR cases, whereas the RR and resident teams’ CPR cases were in-hospital. Nevertheless, this was the first study comparing CPR outcomes across different CPR teams within a single tertiary hospital during the same study period, rather than as a before-and-after study.

## Conclusion

In conclusion, CPR performed by the RR team may result in superior ROSC rates and 10-day survival after ROSC compared to that performed by the resident team. Thus, it may be beneficial if in-hospital CPR is performed by a more experienced team that uses a systematic approach to CPR regardless of the time of day or the day of week. However, our results should be applied with caution owing to the aforementioned limitations, and follow-up studies investigating the optimal composition of CPR teams are recommended.

## Abbreviations

CPR: Cardiopulmonary Resuscitation
EM: Emergency Medicine
ICU: Intensive Care Unit
ROSC: Return of Spontaneous Circulation
RR: Rapid Response
SNUBH: Seoul National University Bundang Hospital
